# A Machine Learning-Based Water Potability Prediction Model by Using Synthetic Minority Oversampling Technique and Explainable AI

**DOI:** 10.1155/2022/9283293

**Published:** 2022-09-20

**Authors:** Jinal Patel, Charmi Amipara, Tariq Ahamed Ahanger, Komal Ladhva, Rajeev Kumar Gupta, Hashem O. Alsaab, Yusuf S. Althobaiti, Rajnish Ratna

**Affiliations:** ^1^Department of Computer Science and Engineering Pandit Deendayal Energy University, Gandhinagar, Gujarat, India; ^2^College of Computer Engineering and Sciences, Prince Sattam Bin Abdulaziz University, Saudi Arabia; ^3^Department of Pharmaceutics and Pharmaceutical Technology, Taif University, Taif 21944, Saudi Arabia; ^4^Addiction and Neuroscience Research Unit, Taif University, Taif 21944, Saudi Arabia; ^5^Department of Pharmacology and Toxicology, College of Pharmacy, Taif University, Taif 21944, Saudi Arabia; ^6^Gedu College of Business Studies, Royal University of Bhutan, Bhutan

## Abstract

During the last few decades, the quality of water has deteriorated significantly due to pollution and many other issues. As a consequence of this, there is a need for a model that can make accurate projections about water quality. This work shows the comparative analysis of different machine learning approaches like Support Vector Machine (SVM), Decision Tree (DT), Random Forest, Gradient Boost, and Ada Boost, used for the water quality classification. The model is trained on the Water Quality Index dataset available on Kaggle. Z-score is used to normalize the dataset before beginning the training process for the model. Because the given dataset is unbalanced, Synthetic Minority Oversampling Technique (SMOTE) is used to balance the dataset. Experiments results depict that Random Forest and Gradient Boost give the highest accuracy of 81%. One of the major issues with the machine learning model is lack of transparency which makes it impossible to evaluate the results of the model. To address this issue, explainable AI (XAI) is used which assists us in determining which features are the most important. Within the context of this investigation, Local Interpretable Model-agnostic Explanations (LIME) is utilized to ascertain the significance of the features.

## 1. Introduction

Whether it is utilized for drinking, household usage, food production, or leisure, safe and readily available water is critical for public health. Improving supplies of water, and also improved management of water resources, might help countries thrive and reduce poverty. There are many reasons why water is deteriorating because in our India there are many industrial areas so the release of pollutants in rivers is the main reason for water deteriorating. There are many other reasons for water deteriorating like people's garbage (plastics), the unwanted things in rivers, their nearest ponds, lakes, and also in sea, and due to plastics and unwanted garbage, there are some toxic occurrences. So, for all these reasons, water is deteriorating nowadays. Contaminated water and inadequate sanitation have been related to diseases such as typhoid, dysentery, polio, cholera, hepatitis, and diarrhea. People are exposed to preventable health dangers due to a lack of, inadequate, or poorly managed water and sanitation facilities. It is especially the case in health facilities, at which water shortage, hygiene, and cleanliness assistance exposes staff and patients to viruses and bacteria. Globally, 15% of people get a virus throughout a stay in the hospital, only with numbers becoming very higher at lower areas. The choice of drinkable water must be decided with great care. Many domain-acknowledgements are required to address this challenge. In this case, this system gets built in that manner to comprehend as a supply of data as much as feasible while retaining generality. In India, however, industrial and home pollutants have contaminated 70% of accessible water.

Approximately 80% of the local population and 20% of the urban population do not have access to clean drinking water. Three-quarters of the nation's children's health issues are infectious diseases and environmental factors, mainly water supply and sanitation. Diarrhea is responsible for 46% of mortality in children under the age of five, with water-related disorders accounting for a large amount of this. According to Ethiopia's Ministry of Health, 6000 children die each day from diarrhea and dehydration.

The contribution of this work is as follows:

- An initial evaluation was carried out on the accessible data in order to filter, normalize, and execute classification algorithms steps to improve water quality in order to find that smallest portion of interest which enables for great level of accuracy at a cheap price. As a result, future identical investigations can avoid costly and time-consuming lab analyses with specific sensors.
- On the dataset, a number of supervised prediction (classification and regression) techniques are chosen as examples. In the context of numerical water quality analysis, the entire approach is proposed.
- In our code, we deploy some models and we get 5 best models for our dataset. So those are XGB (XGBoost), RF (Random Forest), DTC (Decision Tree), ADA (Adaptive Boosting), and SVC (Support Vector Classifier). From these 5 models, we choose the 2 models to find the best accuracy of water. So those 2 models are XGB (XGBoost) and RF (Random Forest). In these 2 models, we obtain 75 to 82% accuracy.

The rest of the work is organized as follows: Section 2 discusses Horton's methodology as well as an overview of the existing methodology.  Section 3 discusses the proposed systems, detailing the steps for accurately gathering data, and preprocesses the gathered data, splitting it up and plots of histograms for various features. The proposed machine learning based model and model evaluation are discussed in Section 4.  Section 5 concludes the work and discusses the future aspects.

## 2. Literature Review

This study looks into the approaches that were used to help solve water quality challenges [1]. In most studies, traditional analyses in the laboratory and data analysis are two types of analyses and utilized to help determine the quality of water, but other studies apply machine learning approaches to help find an optimal solution to the water quality problem.

Consumers' health is being negatively impacted by poor drinking water quality. At least 2 billion individuals used feces-contaminated drinking water around the world, according to reports. Developing accurate decisions about the control and safeguarding of drinking water quality necessitates an awareness of the factors impacting its purity. Potable water quality is typically impacted by the source water's quality, how it is handled before being delivered, how it is distributed, how it is maintained, and how effectively it is filtered at residence. Furthermore, in rural areas and small municipalities, drinking water is frequently drawn straight from wells or retrieved unfiltered from rivers, lakes, and reservoirs. As a result, the purity of the source water is a significant factor affecting the quality of the drinking water. Many developing nations have achieved waterborne disease reduction and the development of safe water supplies a significant public health aim in recent years, and the situation has improved slightly. However, the situation is far from ideal, particularly in rural regions, and even marginally better conditions may be jeopardized by growing water consumption and reduced water availability as a result of population expansion and economic development. It is vital to use a practical and effective drinking water quality evaluation approach to get trustworthy results and make informed decisions.

Many water quality evaluation approaches have been proposed since Horton produced the first Water Quality Index (WQI) in the 1960s [2]. The two indices for determining the general state of drinking source water quality are straightforward, adaptable, and stable, with little sensitivity to input data. Similarly, to give water quality information, we employed the weighted arithmetic WQI approach. These WQIs convert a huge number of variables into a digital number and aid in the comprehension of water quality, making them the most widely used water quality assessment tool, despite significant flaws. Recent water quality assessments used matter element extension analysis (MEEA) and entropy TOPSIS in a wastewater irrigation area and a rapidly urbanizing area, respectively [2]. Both approaches are mathematical, but they are accurate in estimating overall water quality. These water quality evaluation methods, on the other hand, rely on water quality standards for classification. As a result, the most important thing is to create water quality guidelines.

All water utilities shall provide an appropriate, reliable source of greater drinkable water to consumers of price that is proportional to the demands of each water system. To fulfill this goal, its freshwater must be purified and supplied from the greatest source possible sufficiently in order to fulfill regulation and moisture levels sector standards. Consumer acceptance proved treatment procedures, and successful utility management should all be factored in determining the quality of drinking water. The water of high quality is characterized as being free of harmful organisms and biological forms that may be aesthetically unattractive. It is clear and colorless, with no unpleasant odor or flavor. It is free of chemical concentrations that could be detrimental to the body, visually unappealing, or financially destructive. It is also noncorrosive and leaves no excessive or unwanted deposits on water-conveying structures such as pipes, tanks, and plumbing fittings.

Yafra Khan and Chai Soo See [3], in their paper, have used Artificial Neural Network and time series analysis to design a water quality prediction model. Mean Squared Error (MSE), Root Mean Squared Error (RMSE), and Regression Analysis have been used as a part of evaluating the model performance. Dao Nguyen Khoi et al. [4], in their paper, have used 12 machine learning models to estimate the quality of water. Model evaluation was done by using 2 statistics, R2 and RMSE. Umair Ahmed et al. [5] have used supervised machine learning algorithms to estimate the Water Quality Index (WQI). Saber Kouadri et al. [6] used 8 artificial intelligence algorithms to generate Water quality Index prediction. Evaluation of models was done using several statistical metrics, which includes correlation coefficient (R), mean absolute error (MAE), root mean square error (RMSE), relative absolute error (RAE), and root relative square error (RRSE). Jitha Nair and Vijaya M S [7] used various prediction models developed using machine learning and big data techniques using sensor networks.

**Figure 1:**
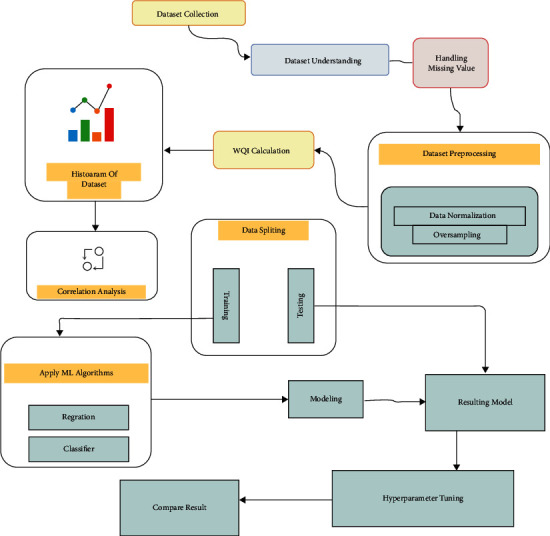
Overview of the proposed system.

Water quality was estimated using traditional machine learning techniques such as XGB (XGBoost), RF (Random Forest), DTC (Decision Tree), Adaptive Boosting (AdaBoost), and SVC, with XGB having the highest accuracy of 83% (XGBoost) [8]. Their work is centered on water quality; all of the factors in the dataset, including hardness, sulfate, solid, trihalomethanes, pH, turbidity, solids, organic carbon, conductivity, are tested according to World Health Organization (WHO) standards [5]. When predicting water quality, using these metrics and comparing them to established values are a significant constraint.  Figure 1 gives a thorough view of the system we have presented.

## 3. Proposed System

The standards used to assess the sustainability of water resources are constantly evaluated as new factors are found. Standards and guidelines for contamination levels in drinking water are being developed by regulatory agencies. In response to the changing criteria, the water supply sector is creating new and improved operating and treatment procedures. All elements that affect water quality, as well as the public health relevance of components and available treatment technology, must be considered when developing drinking water quality guidelines.

The initial task was to find out which factor would give a good indication of the quality of the water. Hardness, sulfate, solid, trihalomethanes, pH, turbidity, solids, organic carbon, and conductivity were chosen as parameters after extensive investigation. Water parameters delve into the logic behind these choices. These measurements provide very little information about how dirty the water is on its own. As a result, the study will take into account the collective behavior of the parameters to produce a legitimate output, which will determine if the water is potable or not.

The second task was to deal with the dataset's missing values. The value of some factors may not be specified while defining the models, and the output may differ as a result. To solve this problem, we have included the mean value of the factor for which data is absent. To train the model efficiently, we first focus on data normalization using Z-score, which is a critical technique in data analysis. To achieve our goal, we appropriately calculate the Water Quality Index (WQI) to analyze water quality. For better representation, we provide a histogram of the dataset, this facilitates for us to observe how our entire dataset is distributed. Then we have applied a correlation technique to determine the ability of two features to change at a constant rate. After that, we have split the entire dataset into two sections: training data and testing data. We used a variety of machine learning algorithms to train the dataset and then compare the models' accuracy. Following the application of those strategies, we employ hyperparameter tuning to evaluate and receive outcomes from our desired model. Finally, we use the accuracy of our suggested models to compare all of the results. As a result, the validity and reliability of our entire study are guaranteed by this approach.  Figure 1 shows the flow diagram of the proposed model.

### 3.1. Data Collection

The dataset used in this approach came from Kaggle's Water Quality Dataset. Some of the metrics employed in this investigation were hardness, sulfate, solid, trihalomethanes, pH, turbidity, solids, organic carbon, and conductivity. The description of all features is given in Table 1.

### 3.2. Data Preprocessing

Data processing is essential in data analysis to increase data quality. Data processing is described as “the collection and manipulation of data components to produce meaningful information.” During this phase, the WQI was derived using the dataset's most essential parameters.

#### 3.2.1. Dealing with Missing Values

There are several methods for replacing missing values. This is the most popular way for resolving numeric column missing values. The mean will not be suitable if there are outliers. Outliers must be dealt with first in such circumstances.

#### 3.2.2. Data Normalization Using Z-Score

The z-score is a popular method of normalization that indicates the number of standard deviations. It is best if it is between -3 and +3. It converts all the values with different scales to the default scale by normalizing the dataset.

To use the z-score to normalize the data, first we need to calculate the variance. For that, we subtracted the mean (*μ*) from the original value (*x*) and added the square of the result and divided it by the total length. Equation (1) represents the variance.

(1) $$\begin{array}{c} \sigma^{2}=\frac{\sum {\left(x_{i}-\mu \right)}^{2}}{N}\text{.} \end{array}$$

Then calculate the standard deviation which is given in (2). For that, take the square root of the variance.

(2) $$\begin{array}{c} \sigma^{2}=\sqrt{\left(\frac{\sum {\left(x_{i}-\mu \right)}^{2}}{N}\right)}\text{.} \end{array}$$

Now, to calculate the Z-score, we subtracted the mean value from an original value and divided it by the standard deviation, resulting in a score which is ideally between 3 and + 3, which displays how many standard deviations a point is above or below the mean as computed by the equation, where *x* represents the original value, µ represents the mean, and *σ* represents the standard deviation. Equation (3) is used to calculate the Z-score.

(3) $$\begin{array}{c} Z=\frac{{\left(x-\mu \right)}^{2}}{\sigma }\text{.} \end{array}$$

#### 3.2.3. Oversampling Using SMOTE

While working with the unbalanced dataset, the problem that might occur is that most machine learning models ignore the minority class, which results in poor performance, but the fact is that the minority class is often the most important class. To overcome this unbalanced dataset problem, we can use the technique of oversampling the minority class of the dataset. In this technique, replication of instances happens in the minority class which is the easiest approach, but these instances do not add much information to the model. Instead of this, we can create new instances by synthesizing old ones. The Synthetic Minority Oversampling Technique, or SMOTE for short, is a type of data augmentation for the minority class.

SMOTE works by identifying adjacent instances in the feature space, drawing a line linking them, and generating a new sample at a position along that line. To be more precise, an instance from the minority class is chosen randomly. Then *k* of the adjacent neighbors (generally *k* = 5) are identified for that example and then a random neighbor is selected. Synthetic instance is generated at a randomly chosen point in feature space between the two instances [9].  Table 2 illustrates the number of samples before and after oversampling.

### 3.3. Water Quality Index (WQI)

The water quality index (WQI) is a single indicator of water quality that is generated utilizing a number of characteristics that are actually representative of the water's quality. Nine parameters are used to calculate the WQI in the traditional way. The formula given below, (4), is used to calculate the WQI.

(4) $$\begin{array}{c} WQI=\frac{\overset{N}{\underset{i=1}{\sum }}q_{i}\times w_{i}\;}{\overset{N}{\underset{i=1}{\sum }}w_{i}}\text{,} \end{array}$$

where N equals the number of attributes and qi equals the quality rating scale for which the formula, in (5), is given as follows:

(5) $$\begin{array}{c} q_{i}=100\times \left(\frac{V_{i}-V_{Ideal}\;}{S_{i}-S_{Ideal}}\right)\text{.} \end{array}$$

And wi is the parameters' standard value, which is calculated by the given equation:

(6) $$\begin{array}{c} w_{i}=\frac{K}{S_{i}}\text{.} \end{array}$$

The proportionality constant (K) can be determined as given in

(7) $$\begin{array}{c} K=\frac{1}{\overset{N}{\underset{i=1}{\sum }}{\;S}_{i}}\text{.} \end{array}$$

### 3.4. Data Visualization

Data distribution of different attributes is shown in Figures 2–10.

### 3.5. Data Analysis

After all of the data processing, different machine learning techniques were used to forecast potability with the fewest number of parameters possible. Before using a machine learning algorithm, various prior processes must be completed, such as analysis of correlation between all the features and splitting of the dataset, to ensure that the data is ready to be fed into the machine learning models.

#### 3.5.1. Correlation Analysis

**Figure 11:**
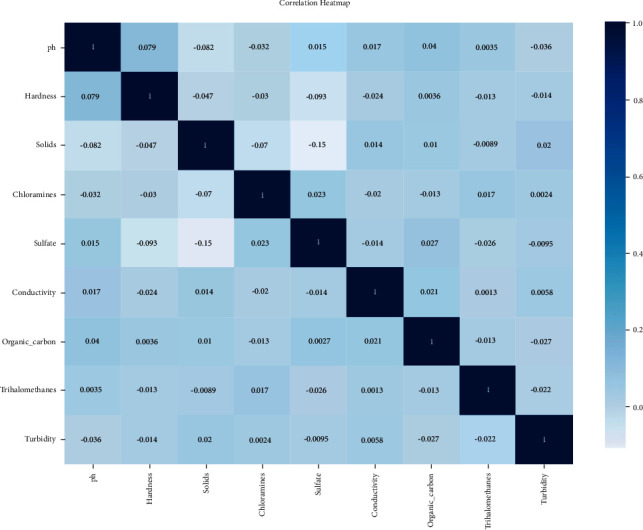
Correlation heatmap.

We used correlation analysis to find possible correlations between all the features in order to find the dependent features using commonly obtainable features. A correlation matrix is a table that displays the correlation coefficients for different characteristics. In a table, the matrix represents all possible value pairs. It is also good for spotting and displaying trends.  Figure 11 shows the correlation between all the features. Now, from the heatmap for correlation analysis, we can observe that the correlation between all the features is very low. That is why we do not have to remove any features from the dataset. Correlations of different features are illustrated in Figure 11.

#### 3.5.2. Data Splitting

In order to train the model, the data must be split, tested with a subset of the data, and computed with accuracy measures to determine the model's performance in the final stage before applying the machine learning model. Training data and test data were created from the dataset. The training data contained 70% of the total dataset and the testing data only contained 30% of the complete dataset. The ML builds a link with the independent and dependent parameters in order to forecast or choose an alternative, and then the test data is taken to determine if the machine learning technique is effective or not.

## 4. Predicting Water Potability Using a Machine Learning Model

### 4.1. Algorithm

Machine learning approaches were used to estimate the water potability in order to meet this aim. We used algorithms for both regression and classification. We employed the following algorithms in our research.

#### 4.1.1. Logistic Regression

A model underlying regression is logistic regression. The approach yields a regression method to forecast the odds that a prescribed data input might well tumble further into category “1.” The sigmoid is used to perform analysis in logistic regression, as illustrated in [10]

(8) $$\begin{array}{c} g\left(z\right)=\frac{1}{1+e^{-z}}\text{.} \end{array}$$

#### 4.1.2. Support Vector Machine Classifier

Supervised learning is the machine preprocessing step that is being used to distinguish and predict the outcome variable. Despite the trouble with regression, classification is the best fit. In (9), x^2^ is rendered to the *Y* axis, although x1 is stretched to the *X* axis. In the scientific fields, pattern recognition, and mentoring segmentation, SVMs are gaining ground [7].

(9) $$\begin{array}{c} f\left(x\right)=\sum \alpha_{j}y_{j}K\left(x_{j}x\right)+b\text{.} \end{array}$$

These are information extraction and penetration testing, simply listing a few.

#### 4.1.3. Decision Tree Classifier

The tree is a monitored form of learning which could be used to counteract obstacles, albeit it is most extensively adopted towards categorization. In a pine classifier, nodes in the network carry collection traits, routes symbolize prior information, and then each node affords the inference [9].

#### 4.1.4. Gaussian Naive Bayes

Categorical data characteristics are acknowledged via Gaussian Naive Bayes, which predicts them all with random variables. To establish a basic model, pretend the input has a distribution function with no dispersion between the components. The characteristics' probability is considered to be shown in the following equation:

(10) $$\begin{array}{c} P\left(\left.x_{i}\right|y\right)=\frac{1}{\sqrt{2\pi \sigma_{y}^{2}}}\exp\;\left(-\frac{{\left(x_{i}-\mu_{y}\right)}^{2}}{2\sigma_{y}^{2}}\right)\text{.} \end{array}$$

#### 4.1.5. Random Forest Classifier

Random Forest is a predictor that estimates the statistics of too many selections applied on discrete clades to optimize a set's anticipated performance. Unlike the decision tree, which is prone to overfitting due to the biasing in the number of nodes in each branch, random forest uses bagging and boosting to combat overfitting and achieve higher accuracy [11].

#### 4.1.6. AdaBoost Classifier

By turning a number of poor learners into strong learners, these methods boost prediction power. Boosting algorithms work on the idea of first building a model on the training dataset and then building a second model to correct the faults in the first model.

#### 4.1.7. Gradient Boost Classifier

In contradiction with AdaBoost, the training context loads are not improved; however, every estimator is prepared by using presidency's errors as symbols. Gradient Boost is a technique that includes Classification and Regression Tree (CART) as the concealer trainee [12, 13].

### 4.2. Measure

In order to evaluate the performance of the model, following metrics are used.

#### 4.2.1. Precision

The proportion of accurately categorized occurrences as in a classifier among all the interpreted contexts is known as precision. Equation (11) is used to compute TP (denoting positive class) while FP is about false alarm in precision.

(11) $$\begin{array}{c} Precision=\frac{TP}{TP+FP}\text{.} \end{array}$$

#### 4.2.2. Accuracy

The proportion of valid simulation provided across all confidence intervals according to the variant is known as accuracy. Equation (12) is used to calculate accuracy, TP conveys true positive, TN signifies true negative, FP reflects false positive, and FN specifies false negative.

(12) $$\begin{array}{c} Accuracy=\frac{TP+TN}{TP+FP+TN+FN}\text{.} \end{array}$$

#### 4.2.3. Recall

The margin for jurisdictions having a certain strong group of individuals willing properly categorized is known as recall. In the formula illustrated in (13) to determine recall, TP accounts as true positive and FN refers to false negative.

(13) $$\begin{array}{c} Recall=\frac{TP}{TP+FN}\text{.} \end{array}$$

#### 4.2.4. F1 Score

Because not everything is enclosed under efficiency and recall elements of validation on their own, as per the formula, we preferred a harmonized average to depict F1 score, 15, which thus encompasses either characteristic and more accurately depicts the total reliability metric. It has a range of 0 to 1. The greater the score is, the more accurate it is.

(14) $$\begin{array}{c} F1\;Score=\frac{2\times Precision\times Recall}{Precision\times Recall}\text{.} \end{array}$$

### 4.3. Result for Algorithms

For creating our classifier and regression model based on the dataset, we used all of the algorithms stated above. However, we were just employing five classifiers, which are the most accurate of all the systems. Random Forest Classifier, Gradient Boosting Classifier, Decision Tree, AdaBoost Classifier, and Vector of assistance are some of the algorithms we used. To evaluate our model, we used hyperparameter tuning on these five classifiers as shows in Table 3.

### 4.4. Hyperparameter Tuning

You will receive granted options available to outlining a framework for a trained model while you are creating it. We might not always realize what optimum solution topology for one fitted model is; hence, we would like to be ready to experience a few more distinct interpretations. We will urge a robot to conduct this analysis to intelligently select the most suitable network model, which is conventional in algorithms. Hyperparameters seem to be the criteria that dictate the system model, whereas parameter tweaking has been the task of evaluating a suitable model infrastructure.

#### 4.4.1. Hyperparameter Tuning

Models might contain a lot of hyperparameters; thus finding the optimum combination of them is a search issue. The following are the two most effective ways for hyperparameter tuning:

*(1) GridSearchCV*. The machine learning model is assessed for a variety of hyperparameter values in the GridSearchCV technique. GridSearchCV is the name given to this method since it searches through a sequence of hyperparameter values to find the ideal incorporation of hyperparameters [14, 15].

*(2) RandomizedSearchCV*. Because it only runs through a predetermined number of hyperparameter settings, RandomizedSearchCV overcomes the shortcomings of GridSearchCV. It travels randomly throughout the grid to discover the optimal collection of hyperparameters. This method eliminates the need for further computation [16, 17].

With the help of GridSearchCV and RandomizedSearchCV [18], we were going to evaluate models for five classifiers: Random Forest Classifier, Gradient Boosting Classifier, Decision Tree, AdaBoost Classifier, and Vector of assistance, and the result of hyperparameter tuning is shown below.

#### 4.4.2. Results of Hyperparameter Tuning

Table 4 shows that, after performing hyperparameter tuning, the accuracy of classifiers improves in terms of precision and best scores, indicating that our model is now evaluated.

### 4.5. Final Model and Results

Water quality is traditionally measured using water quality criteria obtained through time-consuming laboratory examination. We looked at different machine learning approaches for estimating it and discovered various research that used them. The model was evaluated using ten water quality parameters in these experiments.

We were using cross validation to evaluate final model. Cross validation divides the splits of population among *k* segments and propagates over each one, with k-1 fragments serving as well as one substantial number of the training datasets serving as proving ground. A conventional approach to assessing the performance of automation is the k-fold cross-validation procedure. We were using repeated stratified K-fold in which it gives a method for improving a machine learning model's projected performance. Simply repeat the provided mean result throughout all layers by all iterations using the cross-validation routine for several rounds.

The results in Table 5 of cross validation of two classifiers, Random Forest Classifier and Gradient Boost Classifier, which have improved accuracy after hyperparameter adjustment, are shown below. We will now analyze the final model using these two classifiers.

**Figure 12:**
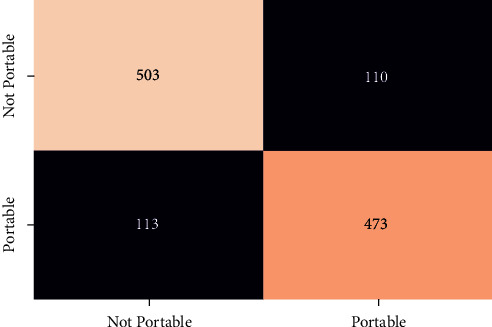
Confusion matrix for RFT.

**Figure 13:**
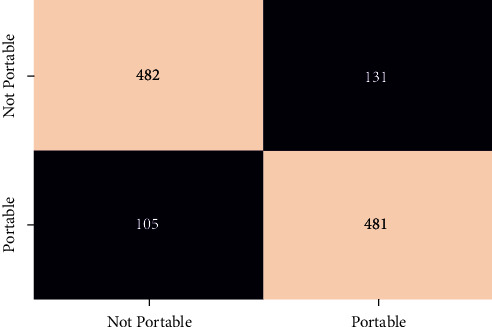
Confusion matrix for XGBoost.

**Figure 14:**
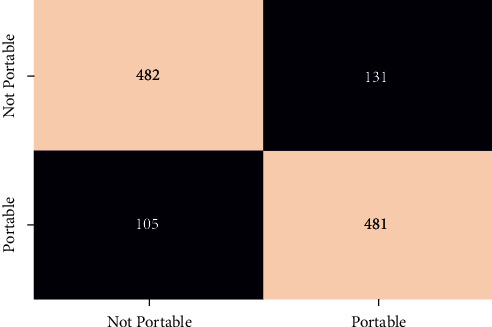
Confusion matrix for Decision Tree.

**Figure 15:**
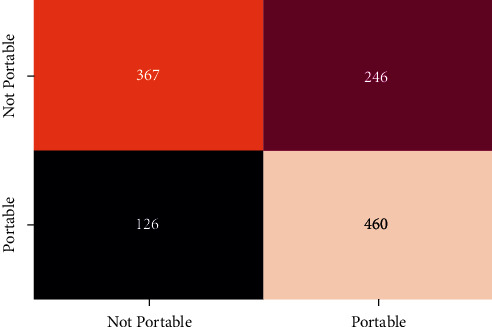
Confusion matrix for ADA.

**Figure 16:**
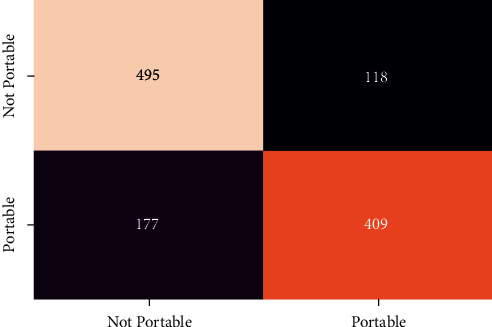
Confusion matrix for SVC.

In order to evaluate the performance of the different classifier confusion matrix and classification report for different classifier is generated. Figures 12–16 show the confusion matrix for RFT, XGBoost, Decision Tree, AdaBoost, and SVC.

Table 6 illustrates the classification report for the different classifier. As shown in Table 6, Random Forest Tree gives better performance among the others machine learning approaches.

One of the key challenges with machine learning and deep learning solutions is lack of transparency. This indicates that it is difficult to describe why any output is being produced by the machine. To address this issue, explainable AI (XAI) is used which finds out how much each parameter contributes to the overall result. The LIME method is used to find the importance of the different features.  Table 7 shows the importance of different features.

As shown in Table 7, sulfate is the feature that contributes the most out of all nine other features. This indicates that the water should not be used for drinking purposes if it contains a high concentration of sulfate.

## 5. Conclusion and Future Work

These are some results which we have been found from the histogram, since there is a difference in the TDS levels. On average, the results are 40 times higher than just the upper threshold for drinkable water. Water samples with acidic and basic pH levels are approximately evenly distributed in the data:

1. In the data that was considered hard of 91.73%
2. The water samples safe for chloramines are only 2.72%
3. The water samples safe for sulfate are only 1.77%
4. The water samples safe for carbon (in 10 ppm) are 90.57%
5. The water samples safe for trihalomethane are 81.62%
6. The water samples safe for utrbidity are 90.42%
7. The correlation coefficients between the features were very low

This study investigated the machine learning performance of approaches as a result of XGB, RF, SVC, ADA, and Decision Trees in predicting the components of a water quality dataset. For this objective, variables in the most well-known datasets, such as pH, hardness, solids, EC, and turbidity, were acquired. The results showed that the applied models performed well in forecasting water quality metrics. Yet, the XGB and RF had the best performance. To make the choosing process more effective, more studies will be carried out. To construct systems which incorporate both suggested along with other strategies and approaches to deep learning.

We must first acquire data for our model data in order to figure out the aspects which would be the most beneficial for forecasting models. We need to conduct data preprocessing in order to rectify any flaws which may appear in our dataset, including such missing values, improperly adjusted data. Then, in order to rate the model, we will split our dataset into two portions: Train and Test. After that, we will utilize our dataset to deploy a machine learning model. After acquiring accuracy, we must improve our model using hyperparameter tuning to achieve the desired accuracy.

In future works, we recommend embedding the research's outcomes into a substantial internet of things system that relies on only the relevant parameter sensors. Based on the real-time statistics provided by the IoT system, the researched algorithms would generate an immediate projection of the water quality. Before water is released publicly for consumption, it would discover toxic water and alert the relevant agencies. Eventually, fewer individuals will consume low-quality water, which would decrease the prevalence of terrible diseases such as typhoid and diarrhea. In this regard, the adoption of a predictive evaluation on the anticipated values would lead to the development of future tools to assist decision and policy makers.

## Figures and Tables

**Figure 2:**
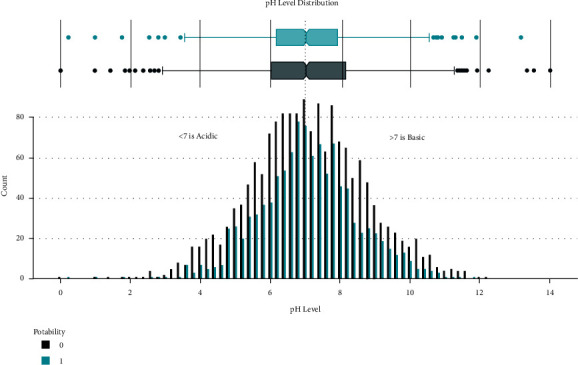
pH value distribution.

**Figure 3:**
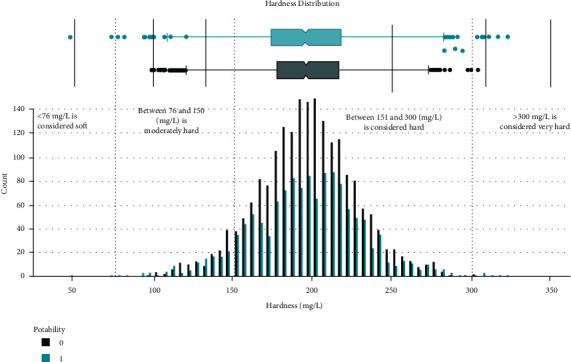
Hardness value distribution.

**Figure 4:**
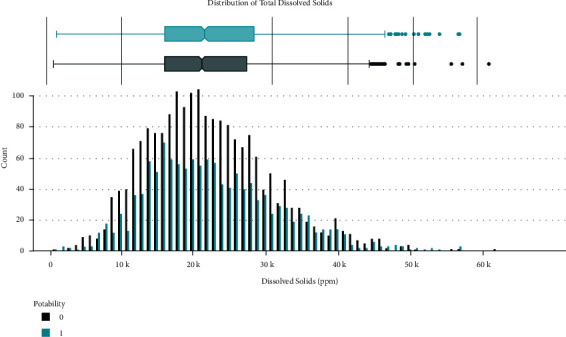
Solid value distribution.

**Figure 5:**
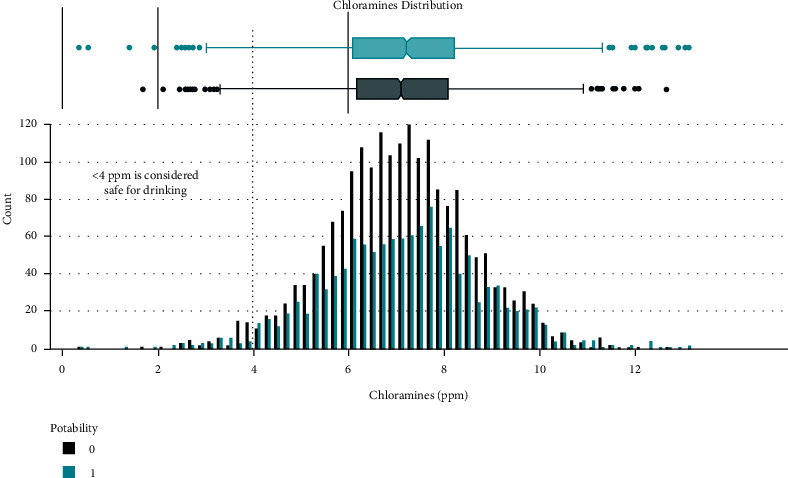
Chloramines value distribution.

**Figure 6:**
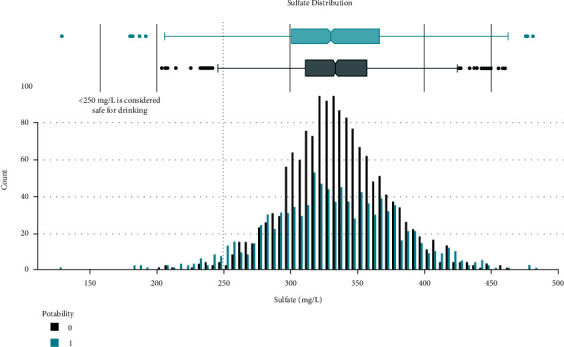
Sulfate value distribution.

**Figure 7:**
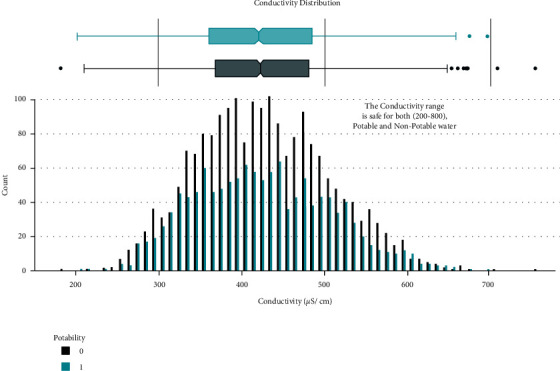
Conductivity value distribution.

**Figure 8:**
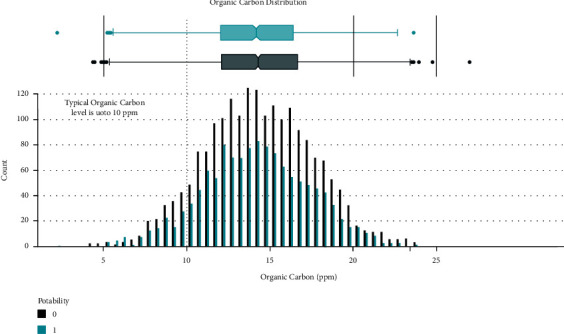
Carbon value distribution.

**Figure 9:**
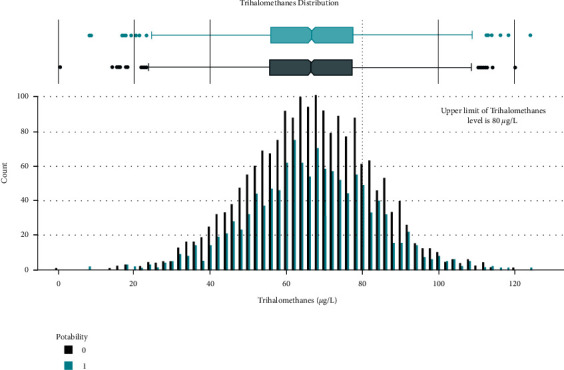
Trihalomethanes value distribution.

**Figure 10:**
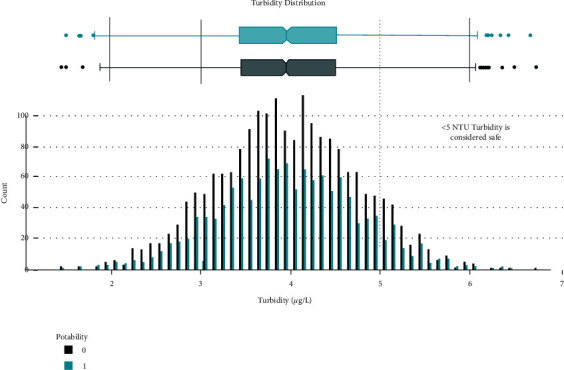
Turbidity value distribution.

**Algorithm 1:**
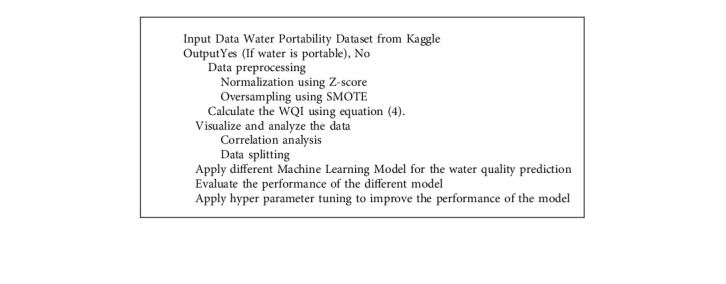
Proposed Model for the Water Portability Prediction.

**Table 1:** Feature description.

Parameters	WHO limits
Ph	6.5–8.5
Hardness	200 mg/L
Solids	1000 ppm
Chloramines	4 ppm
Sulfate	1000 mg/L
Conductivity	400 *μ*S/cm
Organic carbon	10 ppm
Trihalomethanes	80 ppm
Turbidity	5 NTU

**Table 2:** Dataset description before and after oversampling.

	Before oversampling	After oversampling
Not portable	1998	1998
Portable	1278	1998

**Table 3:** Comparative analysis of different classifiers.

Classifiers	Accuracy
Random Forest	0.80
Gradient Boost	0.76
Decision Tree	0.73
Support Vector	0.69
AdaBoost	0.68
Support Vector	0.67
KNeighbors	0.65
BernouliNB	0.61
GaussianNB	0.57
Passive aggressive	0.54
Nearest centroid	0.52
Logistic regression	0.52
Ridge	0.52
Stochastic gradient descent	0.51
Perceptron	0.51

**Table 4:** Hyperparameter tuning.

Model	Accuracy before hyperparameter tuning	Accuracy after hyperparameter tuning	
		Precision score	Best score
Random Forest	0.79	0.83	0.81
Gradient Boost	0.75	0.81	0.81
Decision Tree	0.70	0.71	0.72
AdaBoost	0.69	0.70	0.72
Support Vector	0.68	0.75	0.71

**Table 5:** Cross validation.

Model	Parameters	Accuracy
Gradient Boosting	n_estimators = 500, max_features = log2	0.79
Random Forest	n_estimators = 100, max_features = auto	0.81

**Table 6:** Classification report for different machine learning classifiers.

Model name	Class label	Classification report			
		Precision	Recall	F1- score	Accuracy
RFT	Not portable	0.83	0.80	0.82	0.81
	Portable	0.80	0.81	0.81	

XGBoost	Not portable	0.82	0.79	0.80	0.80
	Portable	0.79	0.82	0.80	

Decision Tree	Not portable	0.74	0.71	0.73	0.73
	Portable	0.71	0.74	0.73	

AdaBoost	Not portable	0.74	0.60	0.66	0.70
	Portable	0.65	0.78	0.71	

SVC	Not portable	0.74	0.81	0.77	0.75
	Portable	0.78	0.70	0.73	

**Table 7:** Importance of different features.

Parameters	Contribution in final output (%)
pH	18
Hardness	9
Solids	9
Chloramines	8
Sulfate	27
Conductivity	7
Organic carbon	8
Trihalomethanes	7
Turbidity	7

## Data Availability

The data that support the findings of this study are available on request from the corresponding author.
